# *Saccharomyces Cerevisiae* Var *Boulardii* CNCM I–1079 Reduces Expression of Genes Involved in Inflammatory Response in Porcine Cells Challenged by Enterotoxigenic *E. Coli* and Influences Bacterial Communities in an In Vitro Model of the Weaning Piglet Colon

**DOI:** 10.3390/antibiotics10091101

**Published:** 2021-09-11

**Authors:** Raphaële Gresse, Juan J. Garrido, Angeles Jiménez-Marín, Sylvain Denis, Tom Van de Wiele, Evelyne Forano, Stéphanie Blanquet-Diot, Frédérique Chaucheyras-Durand

**Affiliations:** 1 Université Clermont Auvergne, F-63000 Clermont-Ferrand, France; raphaele.gresse@inrae.fr (R.G.); sylvain.denis@uca.fr (S.D.); evelyne.forano@inrae.fr (E.F.); stephanie.blanquet@uca.fr (S.B.-D.); 2 Lallemand SAS, F-31702 Blagnac, France; 3 Grupo de Genómica y Mejora Animal, Departamento de Genética, Facultad de Veterinaria, Universidad de Córdoba, 14014 Córdoba, Spain; ge1gapaj@uco.es (J.J.G.); gm2jimaa@uco.es (A.J.-M.); 4 Center for Microbial Ecology and Technology, Ghent University, B-9000 Ghent, Belgium; tom.vandewiele@ugent.be

**Keywords:** in vitro model of colonic microbiota, intestinal cells, piglet, weaning, probiotics, ETEC

## Abstract

Enterotoxigenic *Escherichia coli* (ETEC) is the main infectious agent responsible for piglet post-weaning diarrhea with high mortality rates. Antimicrobials represent the current principal strategy for treating ETEC infections in pig farms, but the occurrence of multi-resistant bacterial strains has considerably increased in the last decades. Thus, finding non-antibiotic alternatives becomes a real emergency. In this context, we investigated the effect of a live yeast strain, *Saccharomyces cerevisiae* var *boulardii* CNCM I-1079 (SB) in an in vitro model of the weaning piglet colon implemented with a mucus phase (MPigut-IVM) inoculated with ETEC and coupled with an intestinal porcine cell line IPI-2I. We showed that SB was able to modulate the in vitro microbiota through an increase in *Bacteroidiaceae* and a decrease in *Prevotellaceae* families. Effluents collected from the SB treated bioreactors were able to mitigate the expression level of genes encoding non-gel forming mucins, tight junction proteins, innate immune pathway, and pro-inflammatory response in IPI-2I cells. Furthermore, SB exerted a significant protective effect against ETEC adhesion on porcine IPEC-J2 intestinal cells in a dose-dependent manner and showed a positive effect on ETEC-challenged IPEC-J2 by lowering expression of genes involved in pro-inflammatory immune responses. Our results showed that the strain SB CNCM I-1079 could prevent microbiota dysbiosis associated with weaning and protect porcine enterocytes from ETEC infections by reducing bacterial adhesion and modulating the inflammatory response.

## 1. Introduction

Enterotoxigenic *Escherichia coli* (ETEC) is the main pathogen responsible of post-weaning diarrhea [1,2,3]. ETEC prevalence has been estimated to 60 to 80 % of piglets between day 1 and 4 post-weaning [4]. The economic losses associated with ETEC infections in swine industry are estimated between 40 and 314 € per sow depending on the country and breeding conditions [1]. To treat post-weaning ETEC infections, antibiotics are, up to date, successfully used but raise major public health concerns due to the wide spread of antibiotic resistance genes in livestock production and globally in the environment in the last decades [1,5]. One of the next challenges of the swine industry is to find non-pharmacological alternatives to reduce the incidence of post-weaning diarrhea while decreasing the use of antibiotics in order to fight against the worrying increase of bacterial resistance towards antibiotics [5]. Probiotics could be potential candidates to help animals resist infections. Probiotics are living organisms that, when administered in adequate amounts, have the potential to confer health benefits to their hosts [6]. If most of the characterized probiotics belong to the bacterial domain such as *Lactobacillus* or *Bifidobacterium* species, yeasts *Saccharomyces cerevisiae* var. *boulardii* (SB) have shown to yield numerous positive effects for animal health, including, for example, better feed efficiency and digestibility, reduced diarrhea, or reduced intestinal inflammation [2,3,4,5]. In livestock production, yeasts have been already commercialized as feed additives which may be able to replace the use of antibiotics as growth promoters [7] but also have been shown to modulate intestinal microbiota, reduce fecal ETEC shedding, decrease diarrhea symptoms, or improve intestinal morphology [7,8,9,10,11,12,13,14]. In vitro trials started to give insights about *S. cerevisiae* and SB anti-infectious properties. In porcine intestinal cell lines challenged with ETEC strains, *S*. *cerevisiae* and SB were both able to inhibit the expression of pro-inflammatory cytokines induced by ETEC infection [12,15]. However, further investigations are needed to unravel the yeast mode of action and beneficial effects as feed additive for weaning piglets.

In this study, we used an in vitro gut fermentation model of the weaning piglet colon (MPigut-IVM) which was recently set-up [16,17] to track the influence of SB CNCM I-1079 on the composition and activity of the resident microbiota challenged with an ETEC F4+ (K88) strain isolated from a diarrheic piglet. Fermentation supernatants were also used to evaluate if the introduction of SB CNCM I-1079 into the in vitro model could affect gene expression of an intestinal porcine cell line. In parallel, we evaluated the direct effects of this live yeast on bacterial adhesion and gene expression of intestinal porcine cells challenged by the ETEC strain.

## 2. Results

### 2.1. SB CNCM I-1079 Inhibits ETEC Adhesion and ETEC-Induced Inflammatory Response on IPEC-J2 Intestinal Cells

**Figure 1:**
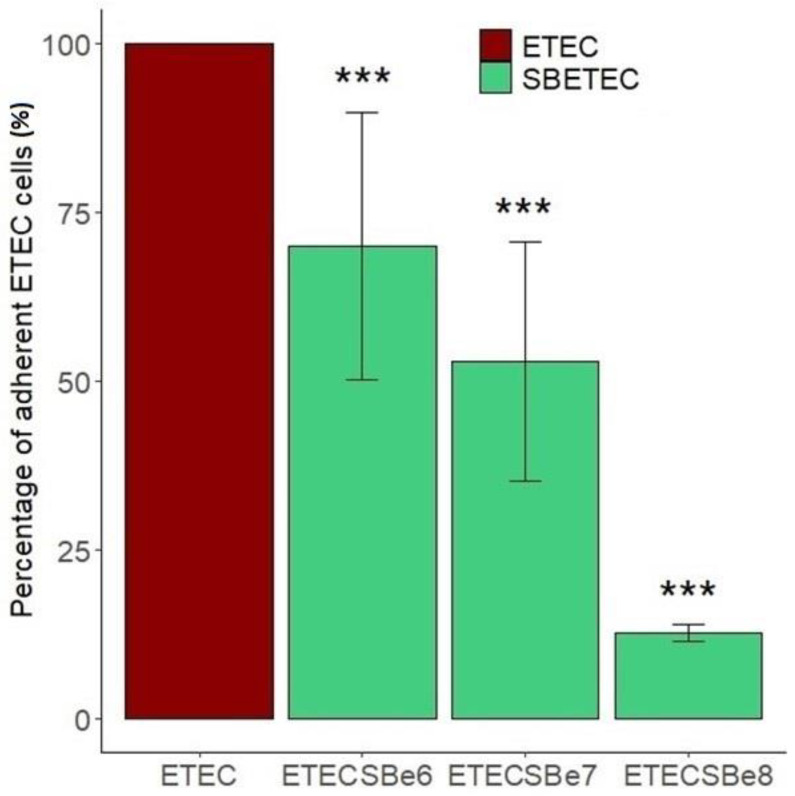
Percentage of adherent ETEC cells on IPEC-J2 intestinal cells pre-treated or not with different doses of SB CNCM I-1079. The concentration of ETEC adherent cells on control IPEC-J2 cells (non-treated with SB yeasts) was normalized to 100% (*n* = 3). The denominations e6, e7, and e8, respectively, correspond to the doses of 10^6^, 10^7^, and 10^8^ CFU/well of SB incubated on the ETEC-challenged IPEC-J2 cells (*p* value codes: *** <0.000).

**Figure 2:**
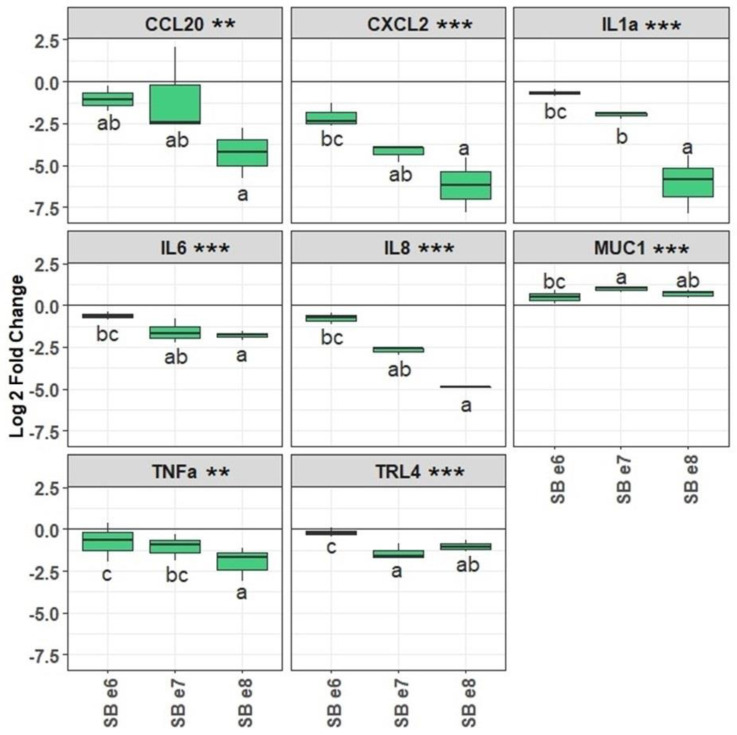
Gene expression of IPEC-J2 cells challenged with the ETEC Ec105 strain and treated or not with different doses of SB CNCM I-1079. Log_2_ fold changes represent the differential gene expression between non-SB treated and SB treated ETEC-challenged IPEC-J2 cells (*n* = 3). Conditions with the same letters are not statistically different from each other, while different letters indicate that the conditions are statistically different from each other. The denominations e6, e7, and e8, respectively, correspond to the doses of 10^6^, 10^7^, and 10^8^ CFU/well of SB incubated with the ETEC-challenged IPEC-J2 cells (*p* value codes: *** <0.000; ** <0.001).

ETEC adherent to IPEC-J2 cells were detected at an average of 1.1 × 10^7^, 4.3 × 10^6,^ and 3.5 × 10^7^ cells/mL, respectively, for the three biological replicates and normalized to 100%. The pre-treatment of IPEC-J2 cells with live SB CNCM I-1079 significantly (*p* < 0.05) reduced ETEC Ec105 adhesion, in a dose-dependent fashion, to reach, respectively, 69 ± 19.7, 53 ± 17.7, and 13 ± 1.2% adhesion for the 10^6^, 10^7^, and 10^8^ SB doses (Figure 1). Moreover, SB also changed the gene expression profile in IPEC-J2 in a dose-dependent manner (Figure 2). For instance, the highest dose of SB significantly reduced the expression of CCL20, CXCL2, IL1a, IL6, IL8, TNFα, or TLR4 genes of, respectively, −4.2 ± 1.5, −6.2 ± 1.6, −6.1 ± 1.7, −1.8 ± 0.3, −4.9 ± 0.1, −2 ± 1, and −1 ± 0.3 Log_2_fold changes comparatively to infected cells which had not been exposed to SB (Figure 2). All pre-treatments slightly, but yet significantly, increased the expression of MUC1 gene.

### 2.2. Live SB CNCM I-1079 Impacts Microbiota Composition and Activity of the Mpigut-IVM Challenged with a Feed Deprivation Stress, a Dietary Change and an ETEC Strain

#### 2.2.1. Effects of SB on ETEC Colonization in the MPigut-IVM

**Figure 3:**
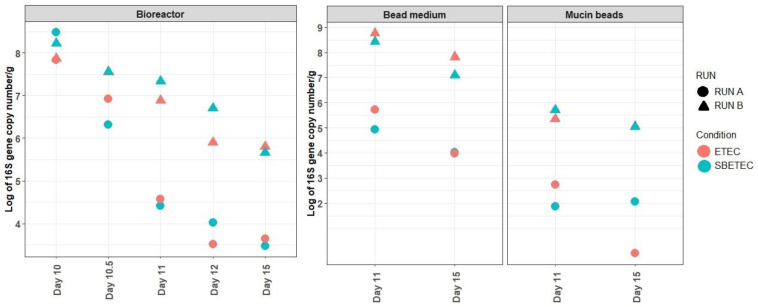
Quantification of the LT enterotoxin gene in the MPigut-IVM during ETEC and SBETEC conditions. Each value is an average of two to three technical replicates.

The quantification of the ETEC LT (labile enterotoxin) gene was used to monitor the presence of the inoculated ETEC strain in the MPigut-IVM and the impact of SB preventive treatment. Overall, the LT gene concentration decreased over time during fermentation whatever the condition, but more quickly in the RUN A where it reached 4 Log_10_ copy numbers/g of content in the bead medium on day 15 compared to the RUN B in which it was maintained at 8 Log_10_ copy numbers/g (Figure 3). 

#### 2.2.2. Effects of SB on Gut Microbiota Activity

**Figure 4:**
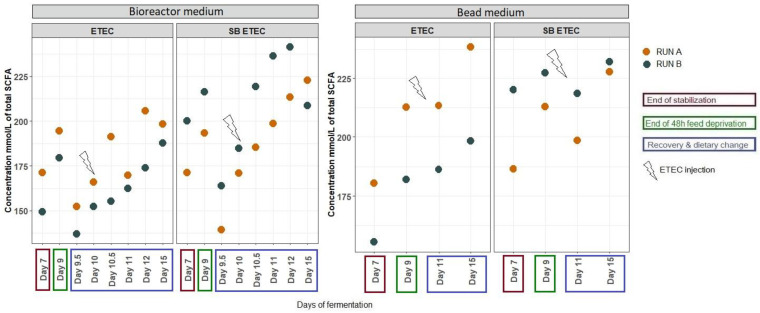
Total SCFA concentration in the MPigut-IVM during ETEC and SBETEC conditions. Each value is an average of two technical replicates.

The average total SCFA concentration was higher in the bioreactor medium in the presence of SB compared to ETEC alone (Figure 4). No difference with SB supplementation was noticed on the proportions of the SCFAs measured in the bioreactor medium nor in the bead medium (Supplementary Figure S1). At day 10, at the time of ETEC inoculation an increase in hydrogen proportion was noticed in the SBETEC condition (13.2%) compared to the ETEC condition (4.4%) as well as a decrease in methane proportion from 6.9% in the ETEC condition to 2.8% in the SBETEC condition but the gas mixture recovered similar profiles later on (Figure S2). 

#### 2.2.3. Effects of SB on Microbiota Composition

**Figure 5:**
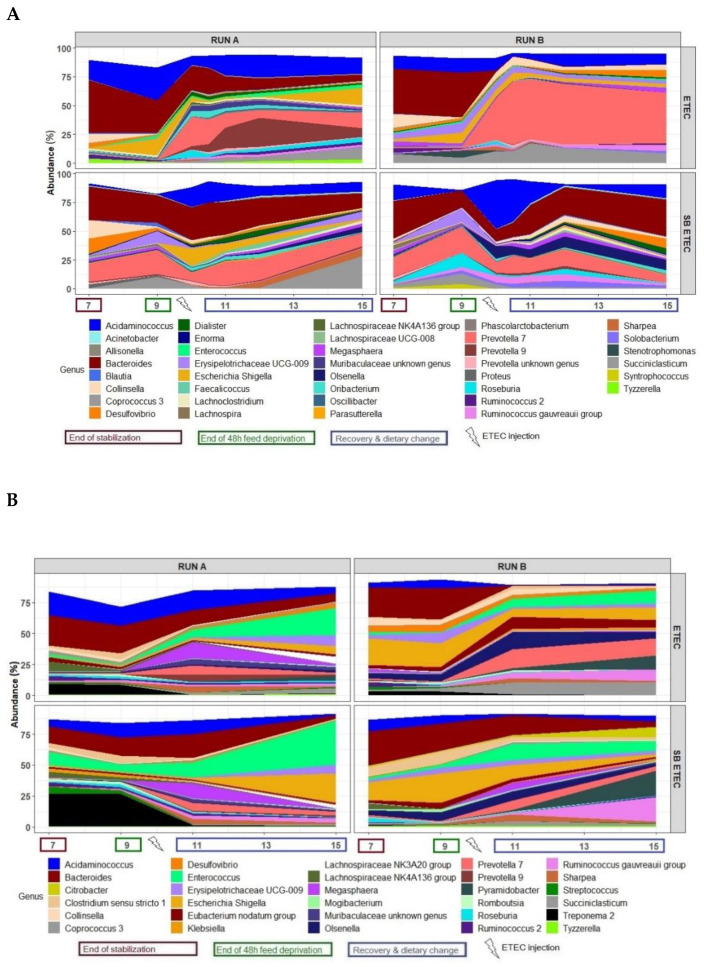
Mean relative abundances of the 30 main bacterial genera in the bioreactor medium (**A**) on the mucin beads (**B**) of the MPigut-IVM during the ETEC and SBETEC conditions.

At the phylum level, the bacterial composition of the in vitro microbiota of the MPigut-IVM challenged with a feed-deprivation stress, a dietary change, and an ETEC strain, and supplemented or not with SB, displayed quite similar profiles in both the bioreactor medium and the mucin beads, with Bacteroidetes and Firmicutes largely dominant, and Proteobacteria, Actinobacteria, Spirochaetes, and Synergistes as minor phyla (Figure S3). No difference was observed in alpha diversity indexes between the ETEC and SBETEC conditions (data not shown). At the family level, the most distinguishable result was the higher relative abundance of *Bacteroidiaceae* and the lower *Prevotellaceae* during the recovery period in the SBETEC compared to the ETEC condition, especially in the bioreactor medium (Figure S4). At the genus level, in the bioreactor medium the main difference observed between SBETEC and ETEC conditions was an evolution of the *Bacteroides*/*Prevotella* ratio (Figure 5A). Indeed, the mean relative abundances of *Bacteroides* and *Prevotella* 7 and 9 genera were, respectively, 16.1% and 13.7% for the ETEC condition while they were 23% and 7.5% in the SBETEC condition from day 10 to day 15. In the bioreactor medium, other differences were noticed such as a higher relative abundance of *Tyzzerella*, *Citrobacter*, *Enterococcus* at day 9, *Eubacterium nodatum* group at day 9, *Escherichia/Shigella* at day 15, *Ruminococcus 2* at days 7 and 15 and *Roseburia* from day 9 to day 15 in SB inoculated fermenters (Figure 5A). On the mucin beads, when SB was distributed daily to the MPigut-IVM and compared to control ETEC bioreactors, we measured higher relative abundances of *Streptococcus*, *Enterococcus*, *Ruminococcus gauvreauii* group, and *Pyramidobacter* at day 15, *Treponema 2* at days 7 and 9, an average higher relative abundance of *Escherichia/Shigella* and a lower relative abundance of *Prevotella 7* after the feed-deprivation period, with quite important discrepancies between runs (Figure 5B).

Regarding archaeal microbiota, SB supplementation promoted the relative abundance of *Methanobrevibacter* at the expense of *Methanosphaera* during the recovery period in particular in the Run B and in the bioreactor medium only (Figure S5).

*Saccharomyces cerevisiae* was detected only in the SBETEC condition, with high but variable concentrations in the bioreactor medium especially after feed-deprivation stress (6 to 10 Log_10_/g), quite high and very stable concentrations (6 Log_10_/g) in the mucin bead medium, and much lower ones on mucin beads (less than 2 Log_10_/g). The concentrations of targeted bacterial and archaeal groups were not impacted by SB supplementation (Figure S6).

### 2.3. SB Live Yeast Supplementation to ETEC-Challenged Mpigut-IVM Leads to Changes in Gene Expression Profile of IPI-2I Intestinal Cells

**Figure 6:**
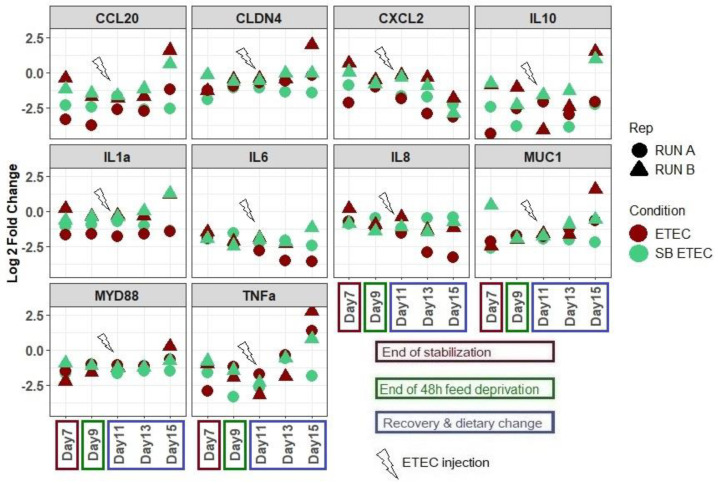
Log_2_ fold changes of IPI-2I cells gene expression when incubated with bead medium supernatants collected from the MPigut-IVM under ETEC and SBETEC conditions. Fold changes are calculated compared to the control condition where IPI-2I cells were incubated with their usual glutamine and FCS complemented DMEM medium. Each value is an average of two technical replicates.

In the presence of supernatants collected from the bead medium of the bioreactors challenged with ETEC and receiving a daily dose of SB, the expression of the genes encoding TNFα, MYD88, CLDN4, and MUC1 in IPI-2I cells was reduced at day 15 when compared to supernatants collected from non-supplemented bioreactors (Figure 6). On the opposite, the IL8 gene expression was increased in the presence of supernatants from the SBETEC condition compared to the ETEC condition at days 13 and 15.

## 3. Discussion

ETEC is the most common cause of diarrhea in farm animals [3,18]. In the last decades, common strategies to fight against infectious post-weaning diarrhea revolved in the use of antibiotics as efficient preventive and curative treatments, however, this massive utilisation is not sustainable due to the increasing dissemination of resistant genes among bacteria [5,19]. More and more investigations now focus on finding non-pharmacological preventive and therapeutic solutions, such as probiotic-based strategies. Several studies highlighted that strains from the *Saccharomyces* genus could be potential candidates for reducing ETEC colonization and signs of infections in weaning piglets [11,20,21]. *Saccharomyces* yeasts showed beneficial impact on several bacterial infections such as the inhibition of *Citrobacter rodentium* adhesion to mice colonic epithelium [22], the inhibition of *Clostridium difficile* toxin production in rat ileum the modulation of ETEC-induced mitogen activated protein pathway on T84 human intestinal cells, and the modulation of pro-inflammatory cellular response in *Shigella* infections via the secretion of proteases degrading pathogen toxins and receptors on T84 intestinal cells [23,24,25]. Another potential mode of action of probiotic yeasts could be the modulation of the intestinal microbiota of piglets at weaning, as already demonstrated in in vivo studies [14]. In the challenged MPigut-IVM with the ETEC F4 porcine strain, the results from two independent biological replicates suggest that the daily supplementation of SB seemed to favour the relative abundance of *Bacteroides* while reducing *Prevotella* members after in vitro simulation of weaning transition [16,17]. As a decrease in relative abundance of *Bacteroidiaceae* in fecal microbiota was previously considered as the onset of post-weaning diarrhea in piglets [26], an increase in *Bacteroides* with SB supplementation may help to limit the intestinal microbiota dysbiosis associated with weaning transition. *Bacteroides* genus is considered as one of the major genera of the core microbiota of mammals particularly known for their strong polysaccharide degradation systems and their beneficial interactions with their host [27]. Studies performed on human microbiota reported that members of *Bacteroides* genus demonstrated their ability to treat intestinal colitis, immune dysfunctions, and metabolic disorders and are considered as potential next-generation of probiotics [27]. SB supplementation to the MPigut-IVM also increased the relative abundance of *Escherichia*/*Shigella* genus, which was not correlated with a higher detection of the LT toxin gene, suggesting that the members of this group would belong to non-pathogenic commensal species. A low relative abundance of commensal *E*. *coli* has been associated with a higher incidence of piglet post-weaning diarrhea [28]. Thus, the increase of commensal *Escherichia*/*Shigella* that we observed with SB treatment could promote ETEC ecological niche filling by commensal *E. coli* and thereby would constitute a protective effect against ETEC colonization in the gut of newly weaned piglets. In agreement with this hypothesis, Bin et al. highlighted that the relative abundance of *E. coli* was increased from 49% to 86% in the jejunum of ETEC-challenged piglets who remained healthy compared to diarrheic individuals [29]. This finding was also supported by Yang et al. who reported that *E*. *coli* was decreased in the fecal microbiota of pre-weaned diarrheic piglets [28]. In addition, the well-known probiotic *E*. *coli* Nissle 1917 has shown efficient suppression of clinical signs against ETEC-induced diarrhea in an infection model of pre-weaned piglets [30]. Deeper analysis of *E*. *coli* strain diversity within in vitro gut microbiota, in particular regarding virulence factors or metabolic traits, would deserve further studies [31]. In addition, there were differences between the two runs in the microbiota responsiveness to the applied stressors and probiotics, that were probably linked to the composition of the initial fecal inoculum, as observed earlier [16,17]. Being able to study individual variability and susceptibility to a given treatment represents an important advantage, nonetheless more research would be needed to confirm the effects of SB treatment with more replicates. Beneficial SCFA producers [32], were also increased by SB treatments in the MPigut-IVM which could help to fight against dysbiosis and ETEC colonization by ensuring an efficient microbiota functionality. A higher mean of total SCFA concentration was indeed detected every day in the SBETEC condition compared to ETEC condition in the bioreactor medium.

The MPigut-IVM proves to be a relevant tool to assess the effects of probiotics. However, a limitation of the developed model is the absence of host-microbiota crosstalk or immune regulations, which are of importance when evaluating the effects of probiotics. Indeed, modifications of gut microbiota composition and activity induced by SB in the MPigut-IVM could have an impact on host cell metabolism or inflammatory response. To try to address this question, in our study we investigated the effects of supernatants from the ETEC-challenged MPigut-IVM bead medium supplemented or not with SB on gene expression in porcine IPI-2I cells. Supernatants from the SB supplemented group seemed to mitigate the expression of TNFα, MyD88, CLDN4, and MUC1 genes that were actually increased in the presence of ETEC, which returned to levels measured in non-ETEC-challenged samples in our previous study [16]. IL8 gene expression was down-regulated by the ETEC-challenged samples, and the SB supplementation seemed to restore basal gene expression of the cells. Although results from the literature about the effects of probiotics on host inflammatory genes are sometimes contradictory [11,33,34,35], in vivo and in vitro studies reported some immunomodulatory effects of *Saccharomyces* species [4]. Wang et al. reported that SB supplementation could reduce the level of TNFα in mice colon [36]. Dogs which received *S. cerevisiae* fermentation products also displayed a lower level of TNFα in their blood [37]. Saegusa et al. reported that, in presence of butyrate, *S*. *cerevisiae* treatment of Caco-2 cells enhanced IL8 production suggesting that some yeasts, hypothetically due to their cell wall component rich in glucans, are able to stimulate the immune system of the host via the increased production of specific cytokines [33]. This last result matches with the higher level of IL8 gene expression that we observed in our IPI-2I cells incubated with MPigut-IVM samples, which also contained butyrate. The slight activation of IL8 gene expression thus does not suggest necessarily that SB provokes inflammation, which would be somewhat contradictory with the decrease in TNFα and MyD88 gene expression. These data also corroborate that the immunomodulatory effects of SB could be influenced by microbial activity (and especially SCFA production, in particular butyrate), however yeast modes of action are far from being fully understood and further studies are needed to understand the complex molecular mechanisms of the dialogue between SB, gut microbiota, and the host. Our results show that the MPigut-IVM can be considered as an effective tool to evaluate the effects of gut microbiota modulation due to pathogens and/or probiotics or other compounds on intestinal epithelial cell response.

After entering orally into the piglet digestive tract, ETEC can attach to enterocytes using fimbrial adhesins [38]. The fimbriae 4 colonization factor is the most prevalent among ETEC responsible for post-weaning diarrhea in piglets [3]. As we wanted to confirm the efficiency of SB against our porcine isolated ETEC strain, IPEC-J2 cells, exhibiting the F4 receptor [39], were pre-treated directly with the active dry yeast SB CNCM I-1079 prior to ETEC challenge. Our results demonstrated a significant protective effect against ETEC F4 adhesion on IPEC-J2 porcine cells, in a dose-response fashion. Additionally, SB also showed a positive effect on ETEC-challenged IPEC-J2 via lowering expression of several genes involved in pro-inflammatory cascade and chemokines involved in immune responses. Then our results showed that the strain SB CNCM I-1079 could protect porcine enterocytes from ETEC infections by reducing bacterial adhesion and modulating the inflammatory response. A few studies investigated the effect of *S. cerevisiae* strains on porcine cell lines and found quite similar results to ours. Zanello et al. showed that on top of its ability to agglutinate with ETEC, the strain *S. cerevisiae* CNCM I-3856 or its culture supernatant could inhibit expression of genes coding for inflammatory cytokines such as IL6, IL8, CCL20, or CXCL2 of ETEC-challenged IPEC-1 and IPI-2I porcine cells [15]. Badia et al. evaluated the role of another yeast strain on ETEC-challenged IPI-2I cells and also noticed a downregulation of TNFα, IL6, CCL2, CXCL8 and CCL20 gene expression [12].

To conclude, our study reported that SB CNCM I-1079 supplementation was able to modulate the MPigut-IVM microbiota and induce differential inflammatory gene expression responses on porcine intestinal epithelial cell experiments, in the context of an ETEC challenge. Then, its use in farms may deserve further attention to be considered as a potential alternative solution to antibiotics to reduce the risk of ETEC infections in piglets around weaning.

## 4. Materials and Methods

### 4.1. MPigut-IVM In Vitro Experiments

#### 4.1.1. Fecal Samples Collection and Treatments

All animals were housed in a conventional pig farm located in the Haute-Loire area of the Auvergne-Rhône-Alpes region in France. Piglets remained with their mother and siblings during the suckling period. None of the piglets had signs of enteric or metabolic disturbances. The animals did not receive any antibiotic in the 27 days prior to feces collection day. Fecal samples from six four-week old healthy male piglets (Landrace x Large White) were collected using sterile bottles and immediately stored under strict anaerobiosis conditions using GENbag anaer gas pack systems (Biomerieux, Marcy L’Etoile, France) and rapidly transported to the laboratory without freezing. Ethical review and approval were not required for the animal study because we collected only fecal material.

#### 4.1.2. MPigut-IVM Parameters

Five hundred mL MiniBio bioreactors (Applikon Biotechnology, Delft, The Netherlands) were equipped with stirrers, ports, and probes, and fecal inocula were prepared as previously described [16,17]. Briefly, to ensure anaerobic conditions at the beginning of fermentations, the same volume of fecal suspension was added per bioreactor to 150 mL of nutritive medium while flushing with O_2_-free N_2_ gas. Afterwards, during the fermentation course, the anaerobic conditions were maintained exclusively through the activity of the resident microbiota and by ensuring the airtightness of the bioreactors. The temperature of the fermentation was set up at 39 °C and maintained inside the bioreactor medium using an incorporated panel heater and in the mucin bead compartment using a hot water bath. pH was maintained at 6.0 and recorded, together with the redox potential, as previously described [16,17]. The fermentation medium was stirred at a constant speed of 300 rpm during the total duration of the experiment. The volume of bioreactors was maintained at a constant value of 200 mL by automatic withdrawal of the fermentation medium. Anaerobic conditions and gas composition were checked every day by analyzing O_2_, CO_2_, CH_4_, and H_2_ using a HP 6890 gas chromatograph (Agilent Technologies, Santa Clara, CA, USA) coupled with a TCD detector (Agilent Technologies, Santa Clara, CA, USA) and two series columns, Molecular Sieve 5A and Porapack Q (Agilent Technologies, Santa Clara, CA, USA).

#### 4.1.3. Mucin Bead Production and Compartment

Mucin from porcine stomach type II (Sigma-Aldrich, Saint-Louis, MO, USA) and sodium alginate (Sigma-Aldrich, Saint-Louis, MO, USA) were used to produce mucin beads of 4 mm diameter, as described previously [16,17]. Briefly, at the beginning of the fermentations, 350 ± 20 mucin beads were introduced in their specific glass compartments. Mucin beads were totally replaced every 48 h to ensure a continuous availability of mucin adherent surfaces. During the time of bead replacement, the medium of the bead compartment was kept under CO_2_ flushing to avoid oxygen entrance.

#### 4.1.4. In Vitro Fermentation Procedures

**Figure 7:**
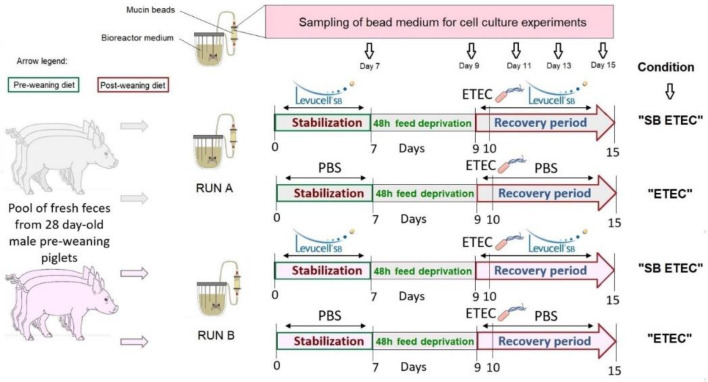
Experimental design of in vitro fermentation procedures and denomination of the MPigut-IVM.

Experiments in the MPigut-IVM were designed as presented on Figure 7. In vitro fermentation procedures aimed to simulate a weaning transition which starts by giving a pre-weaning diet during the first seven days of fermentations. This stabilization period, as previously described, corresponds to the appropriate time to observe a stabilization of microbiota composition and activity inside the MPigut-IVM [16,17]. At day 7, the flow of nutritive medium was interrupted during 48 h to simulate feed deprivation [17]. At day 9, the flow of nutritive medium restarted with a post-weaning diet (defining the recovery period). Pre-weaning and post-weaning diets for MPigut-IVM were prepared to mimic the composition of ileal chyme of piglets as mentioned in Supplementary Table S1. All samplings from day 7 were performed before the start of the feed deprivation period while samplings from day 9 were collected right at the end of the feed deprivation period and before the recovery period. Samples from the bioreactor medium and bead medium were centrifuged at 4 °C, 10,000× *g* for 45 min. Pellets and supernatants were, respectively, stored at −20 and −80 °C until analysis. After collection, mucin beads were gently washed three times in sterile 1X PBS and stored at −20 °C.

#### 4.1.5. ETEC Culture Conditions and Challenge Procedure in The MPigut-IVM

The ETEC strain was an Enterotoxigenic *E. coli* Ec105 (F4, Stb+, East1+, LT+) isolated from a diarrheic piglet (JJ Garrido, personal communication). ETEC Ec105 bacteria were grown at 39 °C overnight (to be consistent with the temperature of the MPigut-IVM) in Luria Bertani (LB) broth (BD Difco, Franklin Lakes, NJ, USA) until OD600nm = 0.6. Cultures were centrifuged at 4 °C, 10,000× *g* for 15 min. The pellet was rinsed using sterile PBS 1X, suspended in 1 mL of sterile PBS and 2 × 10^9^ CFU, corresponding to 10^7^ CFU/mL, were inoculated into the bioreactor medium of the “ETEC” and “SBETEC” conditions at day 10 of the fermentation just before sampling.

#### 4.1.6. SB CNCM I-1079 Supplementation

Every day, except during the feed deprivation period (48h), SB CNCM I-1079 active dry yeast (Levucell SB, Lallemand, Blagnac, France, 2 × 10^10^ CFU/g) was suspended into 1 mL of sterile 1X PBS and inoculated into the MPigut-IVM to reach a final concentration of 10^7^ CFU/mL). This dose was chosen to as close as possible to the concentration of viable yeasts cells entering the piglet colon after a daily supplementation (Lallemand internal data). The “ETEC” condition received a daily dose of 1 mL of sterile 1X PBS as a negative control, except during the feed-deprivation period.

#### 4.1.7. DNA Extraction from MPigut-IVM Samples

Total DNA was extracted from collected samples using the Quick-DNA Fecal/Soil Microbe Miniprep Kit (Zymo Research, Irvine, CA, USA) according to the manufacturer’s instructions. The quality of the eluted DNA was assessed by agarose gel electrophoresis. Extracts were quantified using the Qubit dsDNA Broad Range Assay Kit (Invitrogen, Carlsbad, CA, USA) with a Qubit 2.0 Fluorometer (Invitrogen, Carlsbad, CA, USA). Samples were stored at −20 °C prior to analyses.

#### 4.1.8. Microbial Quantification by qPCR

The list of primer pairs and their optimal conditions used for quantitative PCR of targeted bacterial and archaeal populations in the M-Pigut-IVM are presented in Supplementary Table S2 [40,41,42,43,44]. Real-time PCR assays were performed on a Rotor-Gene Q (Qiagen, Venlo, NL, USA) in 20 µL reactions with QuantiFast SYBR GREEN master mix (Qiagen, Venlo, NL, USA) or Taqman Fast Advanced Master mix (Applied Biosystems, Foster City, California, USA) with the additions of each primer. The 16S rDNA genes were amplified using the following program: 2 min denaturation at 95 °C and 10 min denaturation at 95 °C; 40 and 45 cycles of 20 s at 95 °C and 60 s elongation, and extension at the optimum annealing temperature and, when performing SYBR GREEN based assay, a melting curve step from 60 °C to 95 °C. Each reaction was run in duplicate. The melting curves of PCR amplicons from SYBR GREEN based assays were checked to ensure primer specificity.

#### 4.1.9. MiSeq 16S rDNA Sequencing and Bioinformatic Analysis

The DNA concentration of all samples was measured using the Qubit dsDNA High Sensitivity Assay Kit (Invitrogen, Carlsbad, CA, USA) with a Qubit 2.0 Fluorometer (Invitrogen, Carlsbad, CA, USA) and diluted to 2 ng/µL prior to PCR amplification. The Bacterial V3-V4 region of 16S rDNA and the Archaeal 16S rDNA were respectively amplified with primers 357F 5′-CCTACGGGNGGCWGCAG-3′21 and 805R 5′-GACTACHVGGGTATCTAATCC-3′22 and primers 349F 5′-GYGCASCAGKCGMGAAW-3′ and 806R 5′-GGACTACVSGGGTATCTAAT -3′23. Amplicons were generated using a Fluidigm Access Array followed by high-throughput sequencing on an Illumina MiSeq system (Illumina, San Diego, CA, USA) performed at the Carver Biotechnology Center of the University of Illinois (Urbana, IL, USA). The demultiplexed paired end Illumina sequence reads in the FastQ format were uploaded into the Galaxy instance (v.2.3.0) of the Genotoul bioinformatics platform (http://sigenae-workbench.toulouse.inra.fr, accessed on the 10 September 2021) to be used in the FROGS (Find Rapidly OTU with Galaxy Solution) pipeline [45]. During the FROGS pre-process, sequences were depleted of barcode and the sequences with a non-appropriate length or containing ambiguous bases were removed. Next, reads were clustered into de novo operational taxonomic units (OTUs) using SWARM algorithm [46] with, at first, a denoising step to build a very fine cluster using the minimal distance equal to 1 and, secondly, with an aggregation distance equal to 3. Chimeras were then removed with VSEARCH [47]. Additionally, filters were applied to the OTUs in order to remove singletons [48,49]. The OTUs selected were taxonomically assigned using the Silva release 132 reference database [50].

#### 4.1.10. Quantification of Short Chain Fatty Acids (SCFAs) by Gas Chromatography

SCFAs (acetate, propionate, butyrate, isobutyrate, valerate, isovalerate, and caproate) were quantified in the bioreactor medium and bead medium by gas chromatography, as previously described [16,17]. A mixed-model one-way Anova (lmer and Anova functions) with time (days of fermentation) or conditions as fixed effects and fermentation experiment as a random effect was used to compare the concentration of the main SCFAs between days of fermentation using the R packages lme4 and car.

### 4.2. Porcine Intestinal Cell Line Experiments

#### 4.2.1. Adhesion Assay of ETEC on IPEC-J2 Cells

The IPEC-J2 cell line is a non-transformed intestinal cell line derived from the epithelium of the jejunum of a neonatal non-suckling piglet maintained as a continuous culture [51]. This cell line is generally preferred to IPI-2I cell line for adhesion tests15. IPEC-J2 cells were maintained in Dulbecco’s Modified Eagle Medium (DMEM)/Ham’s F-12 (1:1) medium (InvitrogenTM Life Technologies, Carlsbad, CA, USA) supplemented with 5% Fetal Calf Serum (FCS, PAA Laboratories GmbH, Pasching, Austria). Cells were seeded onto 24-well tissue culture plates at 50,000 cell/well in a volume of 200 µL and grown for 24 h in an atmosphere of 5% CO_2_ at 37 °C to allow for confluence the day of experiment.

Active dry yeast SB CNCM I-1079 (Levucell SB 20, Lallemand SAS, Blagnac, France) was resuspended in DMEM/Ham’s F-12 (1:1) medium (InvitrogenTM Life Technologies, CarlSBad, CA, USA) supplemented with 5% Fetal Calf Serum (FCS, PAA Laboratories GmbH, Pasching, Austria) and rehydrated at 37 °C under agitation for 30 min. ETEC Ec105 strain was grown at 37 °C in LB broth (BD Difco, Franklin Lakes, NJ, USA) until OD600 nm = 0.8–0.9 which corresponds to the concentration of 5 × 10^8^ CFU/mL. ETEC cells were pelleted, washed with sterile PBS and diluted with cell culture medium to reach the concentration of 100 ETEC CFU/ IPEC-J2 cell in a final volume of 100 µL/well. The IPEC-J2 cells on 24 well-plates were first pre-incubated 3 h at 37 °C and 5% CO_2_ with SB at the concentrations of 10^8^, 10^7^, and 10^6^ CFU/well, corresponding, respectively, to 625, 62.5, and 6.25 SB cell/ IPEC-J2 cell, or with 1mL of DMEM medium in triplicates. After 3 h of incubation, IPEC-J2 cells were infected with the ETEC Ec105 strain and incubated for additional 3 h. The entire experiment was run four times. After incubation, for three biological replicates, IPEC-J2 cells were washed three times with sterile 1X PBS and lysed with 1% Triton X-100 (Sigma, St. Louis, MO, USA), and serial dilutions were plated onto LB agar to determine the number of ETEC bacteria adherent to the cells. The last replicate was used for RNA extraction, so IPEC-J2 cells were washed three times with sterile 1X PBS and lysed with 1 mL of NucleoZOL (Machery-Nagel, Hoerdt, France). Lysates were stored at −80 °C prior to RNA isolation.

#### 4.2.2. Incubation of ETEC-challenged MPigut-IVM Samples with an Intestinal Porcine Cell Line

The IPI-2I cell line derived from the ileum of an adult male pig and immortalized by transfection with an SV40 plasmid (pSV3-neo) [52], was used for this co-incubation assay due to its potential similarity with colonic enterocytes. IPI-2I cells were maintained in DMEM/Ham’s F-12 (1:1) (InvitrogenTM Life Technologies, Carlsbad, CA, USA) supplemented with 10% Fetal Calf Serum (FCS, PAA Laboratories GmbH, Pasching, Austria) and 4 mM L-glutamine (Sigma, St. Louis, MO, USA). Cells were seeded onto 48-well tissue culture plates at 25,000 cell/well in a volume of 200 µL and grown 24 h in an atmosphere of 5% CO_2_ at 37 °C to allow for confluence at the day of experiment. Supernatants from fermentation medium and bead medium collected from days 7, 9, 11, 13, and 15 of fermentation were filtered using 0.2 µm sterile Minisart syringe filters (Sartorius, Göttingen, Germany) and 30 times diluted with DMEM (10% Fetal Calf Serum and 4mM L-glutamine). The diluted samples were added in duplicates to confluent monolayers of IPI-2I cells in 48-well plates, as described above. Plates were incubated during 2 h at 37 °C, 5% CO_2_. At the end of the incubation, supernatants were removed and IPI-2I cells were lysed by the addition of 500 µL of NucleoZOL (Macherey-Nagel, Hoerdt, France).

#### 4.2.3. RNA Isolation from Cell IPEC-J2 and IPI2-I lysates

Total cellular RNA was extracted from IPEC-J2 and IPI-2I lysed cells following the NucleoZOL user manual (Macherey-Nagel, Hoerdt, France). The TURBO DNA-freeTM kit (Applied Biosystems, Foster City, CA, USA) was used according to the manufacturer’s instructions to prevent DNA contamination. Purity and quality of the RNA extracts were controlled on 1% agarose gels. RNAs were then quantified using a Nanodrop 1000 spectrophotometer (Thermo Fisher Scientific, Waltham, MA, USA) by measuring the optical density at 260 nm.

#### 4.2.4. RT-qPCR on Porcine Intestinal Cell RNA Extracts

Reverse transcription was performed using the qScript cDNA Synthesis Kit (Quantabio, Beverly, MA, USA). Briefly, 350 ng of RNA per sample was added to 5 µL of sScript Reaction Mix (5x) and 1 µL of qScript Reverse Transcriptase in a final volume of 15 µL. The reverse transcription mix was incubated for 5 min at 22 °C, 30 min at 42 °C and 5 min at 85 °C. The synthetized cDNAs were stored at −20 °C until used. Quantification of genes listed in Supplementary Table S3 was carried out using a QuantStudioTM 12K Flex Real-Time PCR system (Applied Biosystems, Foster City, CA, USA). The cyclophilin A and β-actin genes were used as reference genes. PCR reactions were carried out in 96-well plates using 3 µL of 5x HOT FIREPol^®^ EvaGreen^®^ qPCR Mix Plus (ROX) (Solis BioDyne, Tartu, Estonia), 0.4 µL of forward and reverse primers (Supplementary Table S3) [53], 9.2 µL of milliQ water and 2 µL of cDNA. Ten-fold dilution series of each primer pair were used as standard curves to determine primer efficiencies. Real time PCR efficiencies were calculated according to the equation: E = 10 (−1/slope). The appropriate reference gene and the Log2 fold change for each gene were determined by GenEx software (http://genex.gene-quantification.info/, accessed on the 10 September 2021). A mixed-model one-way Anova (lmer and Anova functions) with the different conditions as a fixed effect and replicates as a random effect was used using the R packages lme4 and car (RStudio software version 1.0 with R software version 3.5.1, R Development Core Team).

## Data Availability

The data generated and/or analysed during the current study will be available in the BioProject database repository, https://www.ncbi.nlm.nih.gov/bioproject/ (accessed on 1 September 2021).

## Supplementary Materials

The following are available online at https://www.mdpi.com/article/10.3390/antibiotics10091101/s1, Figure S1: Relative abundance of SCFA during the ETEC and SBETEC conditions; Figure S2: Mean relative abundances of gas produced by fermentation activity of the microbiota inhabiting the MPigut-IVM under ETEC and SBETEC conditions (*n* = 2); Figure S3: Relative abundances of the 5 main bacterial phyla in the bioreactor medium (A) and mucin beads (B) of the MPigut-IVM during the ETEC and SBETEC conditions; Figure S4: Relative abundance of the 15 main bacterial families in the bioreactor medium (A) and mucin beads (B) of the MPigut-IVM during the ETEC and SBETEC conditions; Figure S5: Relative abundance of the archaeal genera in the bioreactor medium (A) and mucin beads (B) of the MPigut-IVM during the ETEC and SBETEC conditions; Figure S6: Quantification of bacterial and archaeal groups by qPCR in the bioreactor medium (A), mucin beads (B) and bead medium (C) of the MPigut-IVM during the ETEC and SBETEC conditions (*n* = 2); Table S1: Composition of the fermentation medium introduced in the M-Pigut-IVM and mimicking the composition of ileal chyme of piglets when ingesting a pre- or post-weaning diet; Table S2: Primers and probes used for quantification of bacteria and methanogenic archaea in the M-Pigut-IVM; Table S3: Primer sets used for quantification of gene expression in porcine epithelial cells.
