# Supplementary material for: Saccharomyces Cerevisiae Var Boulardii CNCM I–1079 Reduces Expression of Genes Involved in Inflammatory Response in Porcine Cells Challenged by Enterotoxigenic E. Coli and Influences Bacterial Communities in an In Vitro Model of the Weaning Piglet Colon
Source: Antibiotics (Basel). 2021 Sep 11;10(9):1101. doi: 10.3390/antibiotics10091101 (PMC8467900; doi:10.3390/antibiotics10091101)
Supplement: Supplementary file 1 [file antibiotics-10-01101-s001.zip › antibiotics-1360317-supplementary.pdf]

SUPPLEMENTARY MATERIAL

Supplementary Table S1. Composition of the fermentation medium introduced in the M-Pigut-IVM and mimicking the composition of ileal chyme of piglets when ingesting a pre- or post-weaning diet.

	**Pre-weaning diet**	**Post-weaning diet**
**Concentration**	g/L	g/L
Wheat starch	5	5
Corn starch	0	6
Pectin	2	2
Arabinogalactan	2	2
Cellulose	2	3
Soy protein isolate	10	12
Peptone from potatoes	0	5
Tryptone from casein	4.5	1.5
Mucin from porcine stomach type II	2	2
Whey powder	5	2
$FeSO_4$, $7H_2O$	0.005	0.005
L-cysteine HCl monohydrate	0.80	0.80
Porcine bile extract	0.40	0.40
KH_2PO_4	0.5	0.5
$NaHCO_3$	1.5	1.5
Hemin from porcine	0.005	0.005
NaCl	4.5	4.5
KCl	4.5	4.5
$MgSO_4$ anhy. (120.37 g mol^{-1})	0.64	0.64
$CaCl_2$ $2H_2O$ (147.02 g mol^{-1})	0.15	0.15
$MnCl_2$ $4H_2O$ (197.91 g mol^{-1})	0.20	0.20

Supplementary Table S2. Primers and probes used for quantification of bacteria and methanogenic archaea in the M-Pigut-IVM.

Target	Type	Sequences	References	Concentration	Annealing temperature
***Escherichia coli*/ *Shigella* group**	Taqman	5’-CAT GCC GCG TGT ATG AAG AA-3’<br>5’-CGG GTA ACG TCA ATG AGC AAA-3’<br>(6-FAM)-5’-TAT TAA CTT TAC TCC CTT CCT CCC CGC TGA A-3’(TAMRA)	40	300 nM<br>300 nM<br>100 nM	61°C
**Total Bacteria**	SYBRGREEN	5’-ACT CCT ACG GGA GGC AG-3’<br>5’-GTA TTA CCG CGG CTG CTG-3’	41	500 nM<br>500 nM	61°C
**Methanogenic archaea**	SYBRGREEN	5’-GAG GAA GGA GTG GAC GAC GGTA-3’<br>5’-ACG GGC GGT GTG TGC AAG-3’	42	500 nM<br>500 nM	61°C
S. cerevisiae	SYBRGREEN	5’- AGGAGTGCGGTTCTTTG -3’<br>5’- TACTTACCGAGGCAAGCTACA -3’	43	5 µM	60°C
**Labile enterotoxin (LT)**	SYBRGREEN	5’- GGC GTT ACT ATC CTC TCT AT<br>3’—TGG TCT CGG TCA GAT ATG T	44	500 nM<br>500 nM	55°C

Supplementary Table S3. Primer sets used for quantification of gene expression in porcine epithelial cells

Target	Gene	Sequence 5’ 3’	Annealing temperature	Reference
Cyclophilin A	*Cyclophilin-A*	CCT GAA CAT ACG GGT CCT G<br>AAC TGG GAA CCG TTT GTG TTG	57	Dr. J.J. Garrido
Beta actin	*β-actin*	CAGGTCATCACCATCGGCAACG<br>GACAGCACCGTGTTGGCGTAGAGGT	57	Dr. J.J. Garrido
Chemokine (C-C motif) ligand 20	*CCL20*	ACTT TGA CTG CTG CCT CCG ATA<br>TGC ATT GAT GTC ACA AGC TTC A	54	Dr. J.J. Garrido
Claudin 4	*CLDN4*	TAT CAT CCT GGC CGT GCT A<br>CAT CAT CCA CGC AGT TGG T	57	53
Chemokine (C-X-C motif) ligand 2	*CXCL2*	GGA TAG CAC GCT GTA CCA TC<br>ACT GTC TCA ATA AAT AAC AAC CGA C	57	Dr. J.J. Garrido
Interleukin 10	*IL10*	CAG ATG GGC GAC TTG TTG<br>ACA GGG CAG AAA TTG ATG AC	57	Dr. J.J. Garrido
Interleukin 1a	*IL1a*	AAC GAA GAC GAA CCC GTG TTG CT<br>GGT CTC ATC TTT GAT GGT TTT GG	57	Dr. J.J. Garrido
Interleukin 6	*IL6*	TGG CTA CTG CCT TCC CTA CC<br>CAG AGA TTT TGC CGA GGA TG	57	Dr. J.J. Garrido
Interleukin 8	*IL8*	TTC GAT GCC AGT GCA TAA ATA	57	Dr. J.J. Garrido

		CTG TAC AAC CTT CTG CAC CCA		
Mucin 1	*MUC1*	CCC TGG CCA TCA TCT ATG TC	56	53
		TGC CCA CAG TTC TTT CGT C		
Myeloid differentiation primary response 88	*MYD88*	TGG TGG TGG TTG TCT CTG ATG A	57	Dr. J.J. Garrido
		TGG AGA GAG GCT GAG TGC AA		
Tumor Necrosis Factor alpha	*TNF-α*	CGC CCA CGT TGT AGC CAA TGT	57	Dr. J.J. Garrido
		CAG ATA GTC GGG CAG GTT GAT CTC		

Supplementary Figure S1. Relative abundance of SCFA during the ETEC and SBETEC conditions.

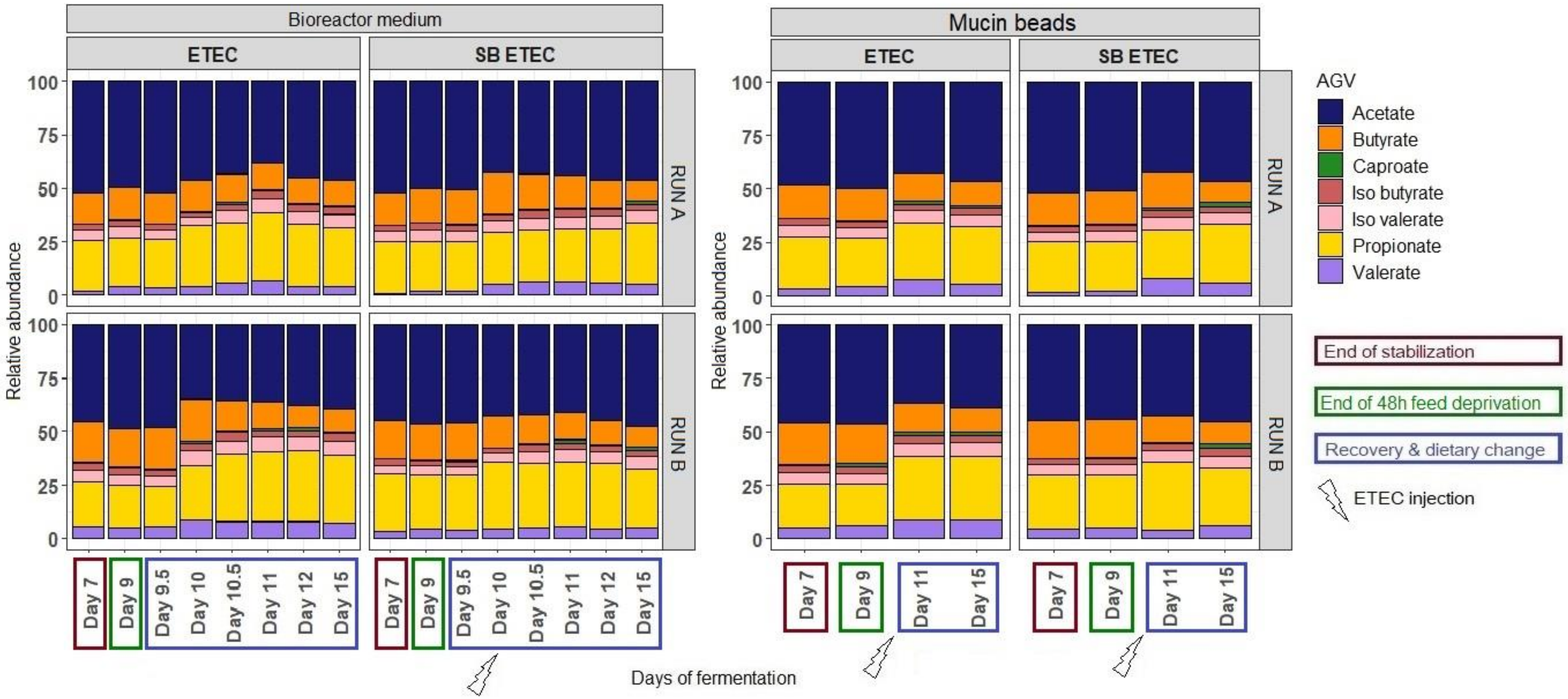

Supplementary Figure S2. Mean relative abundances of gas produced by fermentation activity of the microbiota inhabiting the MPigut-IVM under ETEC and SBETEC conditions (n = 2).

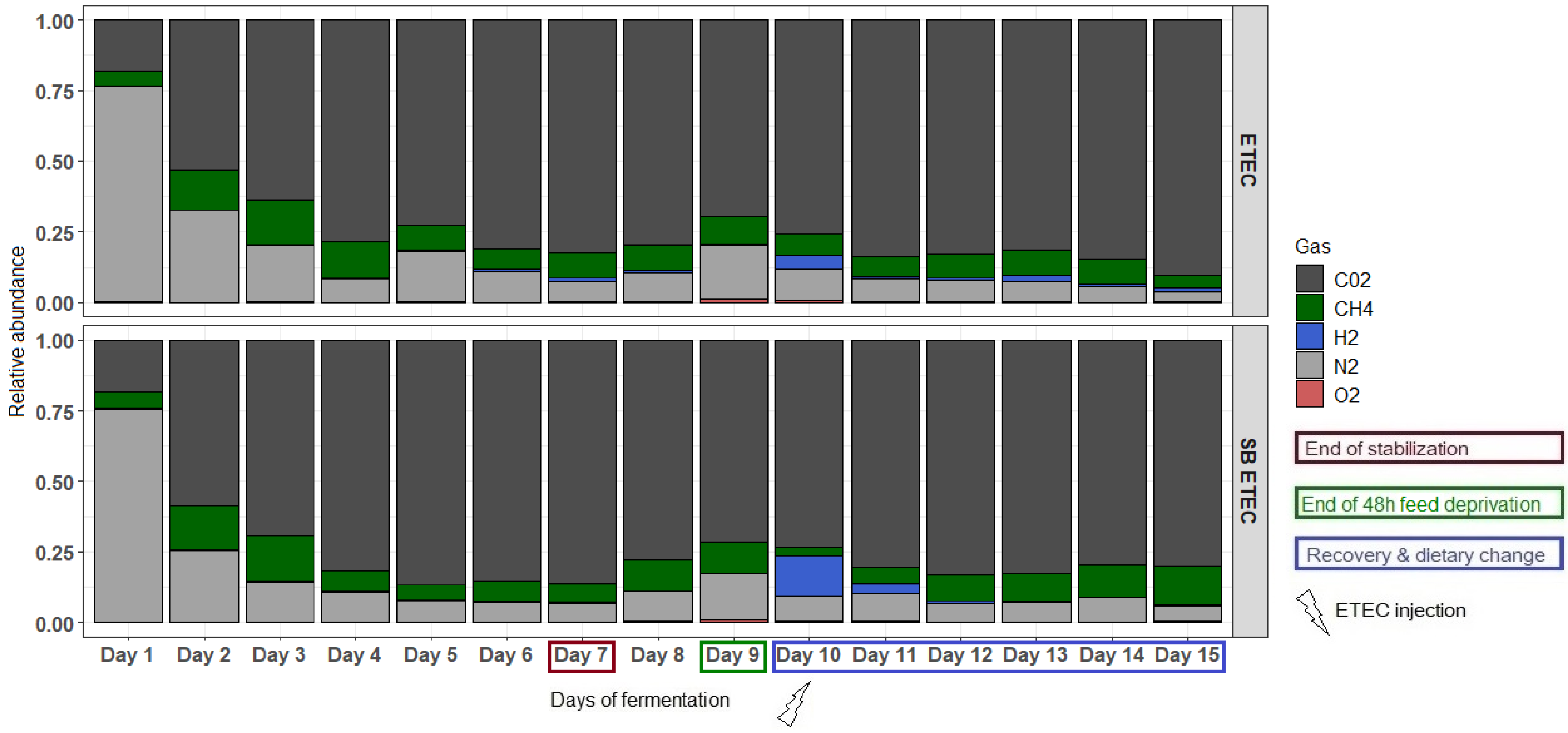

Supplementary Figure S3. Relative abundances of the 5 main bacterial phyla in the bioreactor medium (A) and mucin beads (B) of the MPigut-IVM during the ETEC and SBETEC conditions.

A

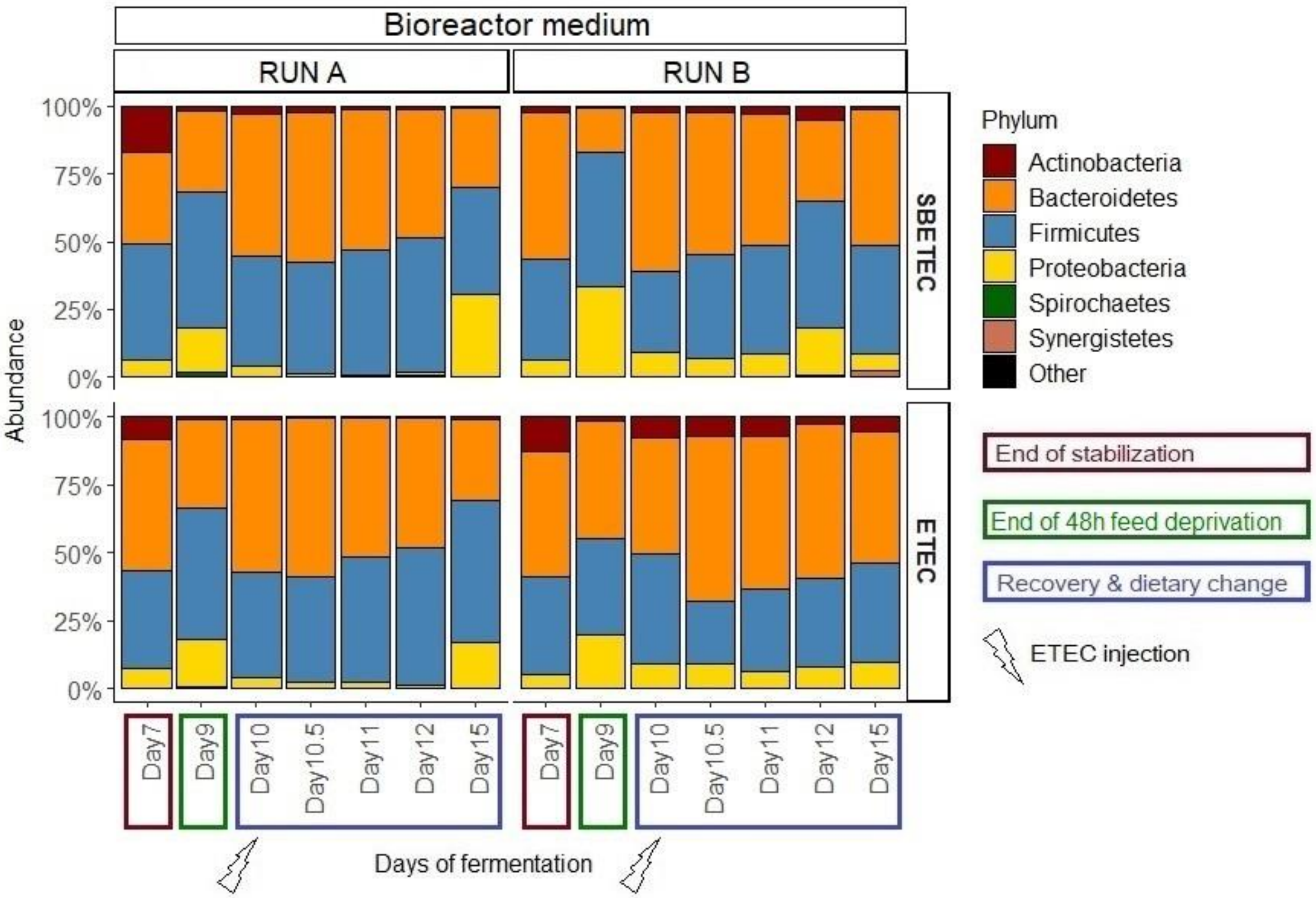

B

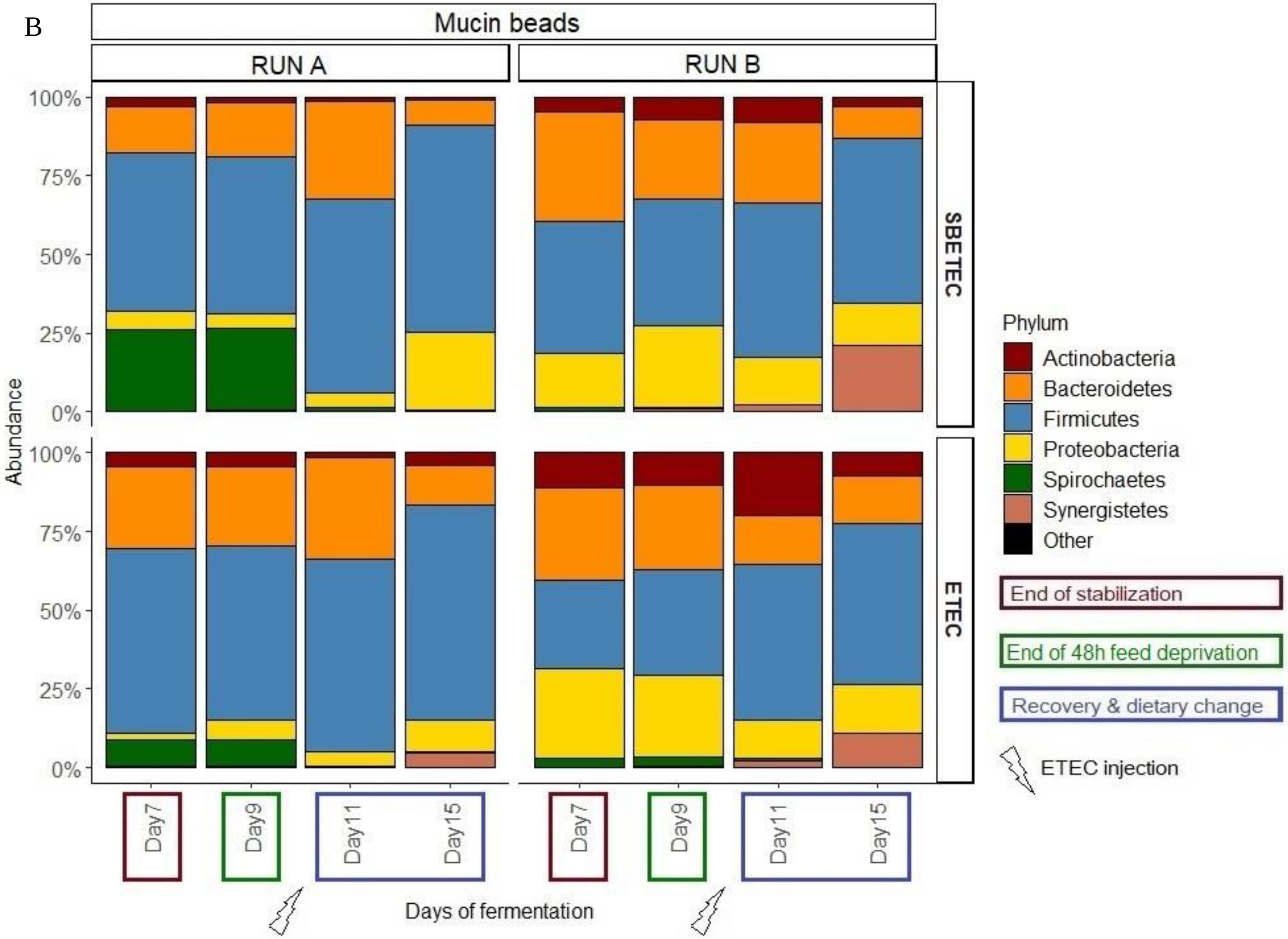
Mucin beads
RUN A
RUN B
SBETEC
ETEC
Abundance
100%
75%
50%
25%
0%
Day7
Day9
Day11
Day15
Days of fermentation
Phylum
Actinobacteria
Bacteroidetes
Firmicutes
Proteobacteria
Spirochaetes
Synergistetes
Other
End of stabilization
End of 48h feed deprivation
Recovery & dietary change
ETEC injection

Supplementary Figure S4. Relative abundance of the 15 main bacterial families in the bioreactor medium (A) and mucin beads (B) of the MPigut-IVM during the ETEC and SBETEC conditions.

A

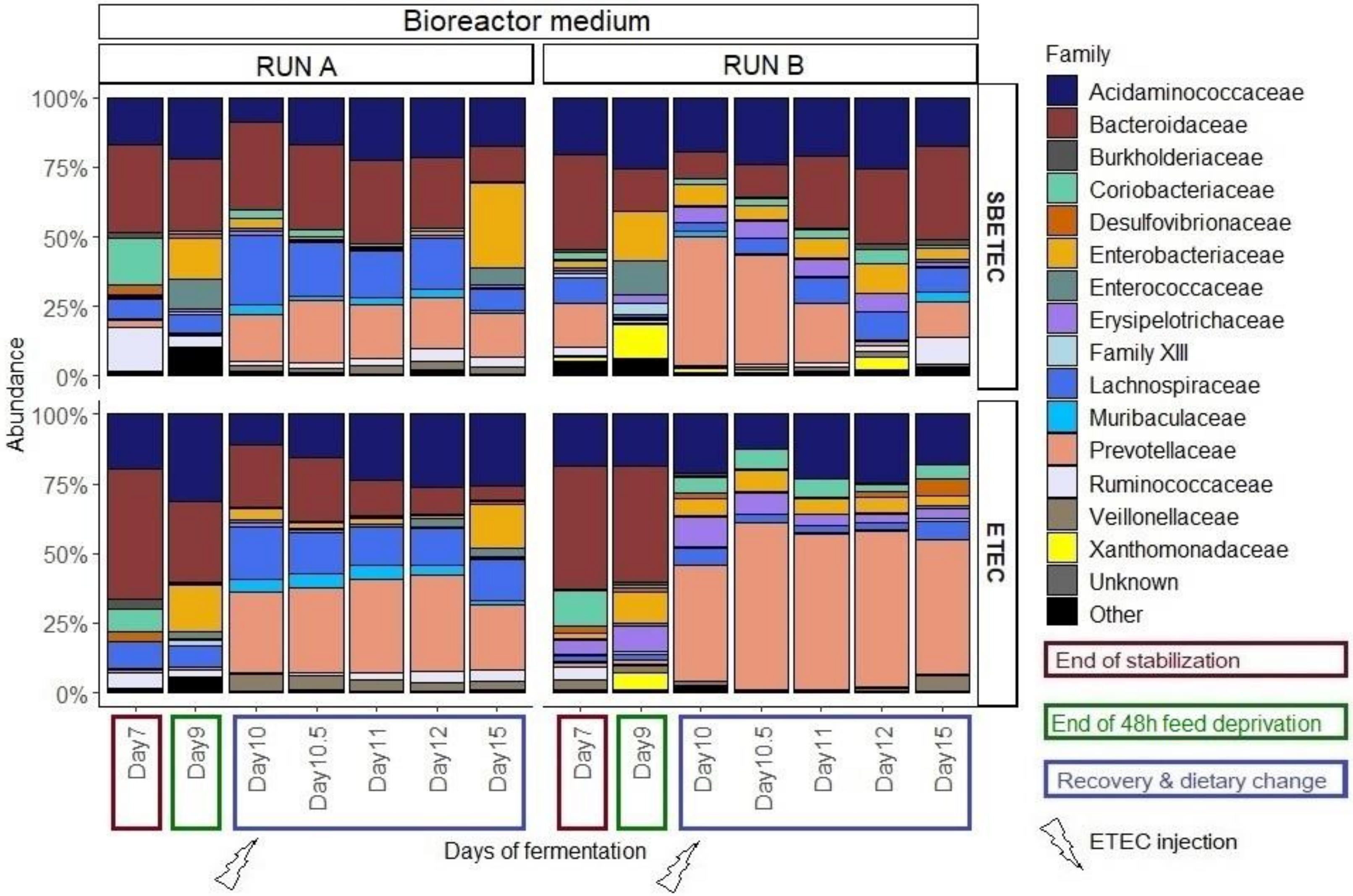

B

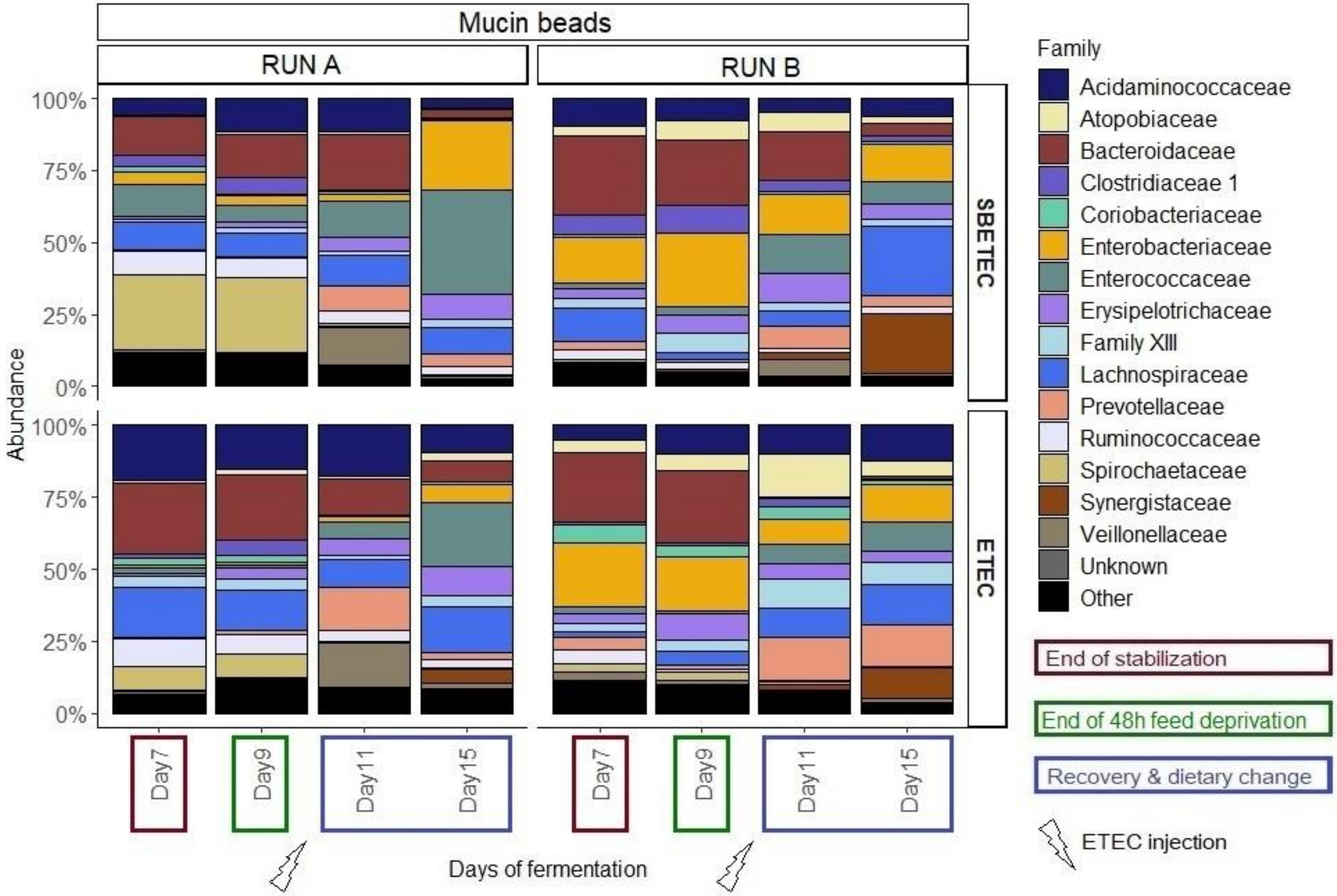

Mucin beads
RUN A
RUN B
100%
75%
50%
25%
0%
Abundance
SBETEC
ETEC
Day7
Day9
Day11
Day15
Days of fermentation
Family
Acidaminococcaceae
Atopobiaceae
Bacteroidaceae
Clostridiaceae 1
Coriobacteriaceae
Enterobacteriaceae
Enterococcaceae
Erysipelotrichaceae
Family XIII
Lachnospiraceae
Prevotellaceae
Ruminococcaceae
Spirochaetaceae
Synergistaceae
Veillonellaceae
Unknown
Other
End of stabilization
End of 48h feed deprivation
Recovery & dietary change
ETEC injection

Supplementary Figure S5. Relative abundance of the archaeal genera in the bioreactor medium (A) and mucin beads (B) of the MPigut-IVM during the ETEC and SBETEC conditions.

A

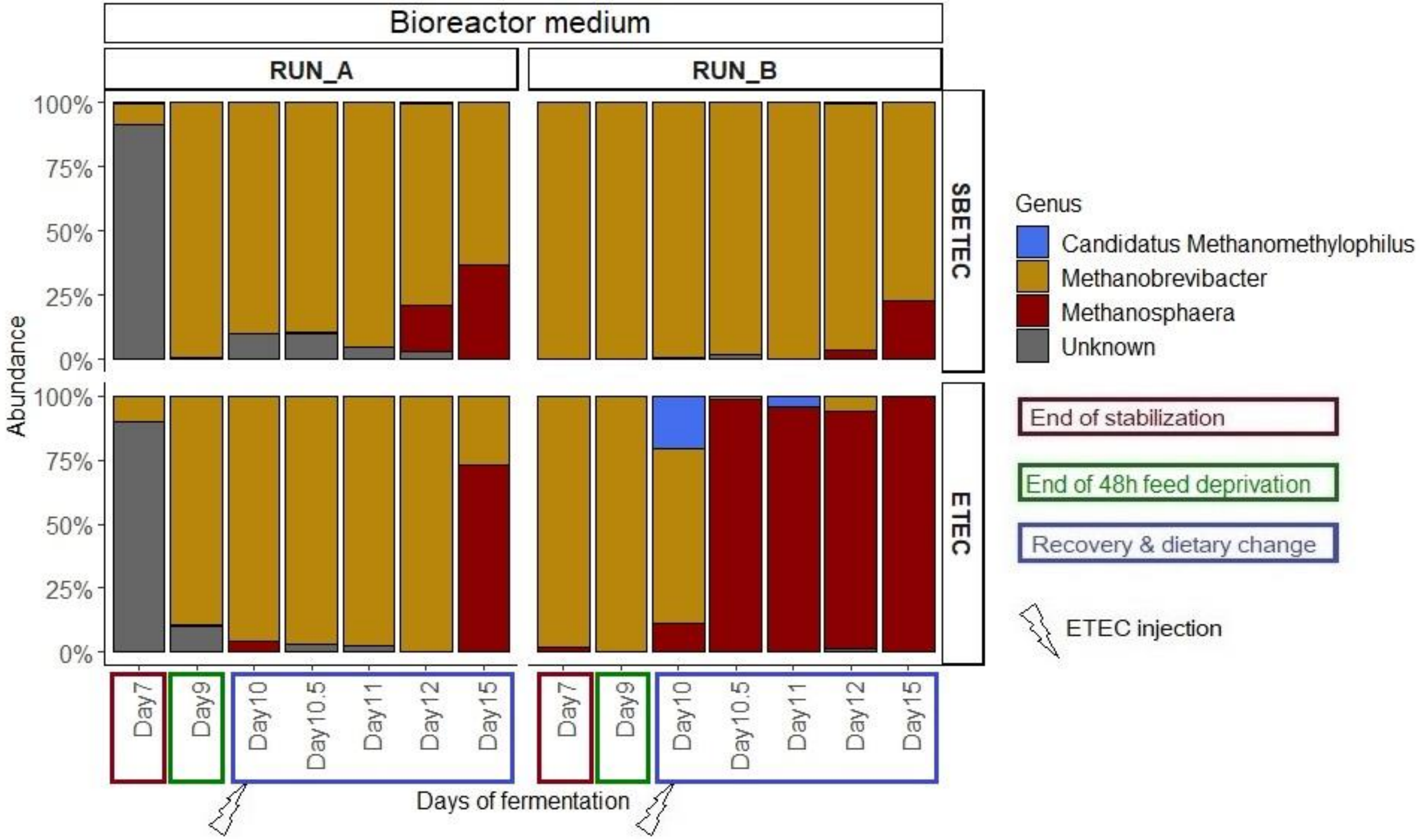

B

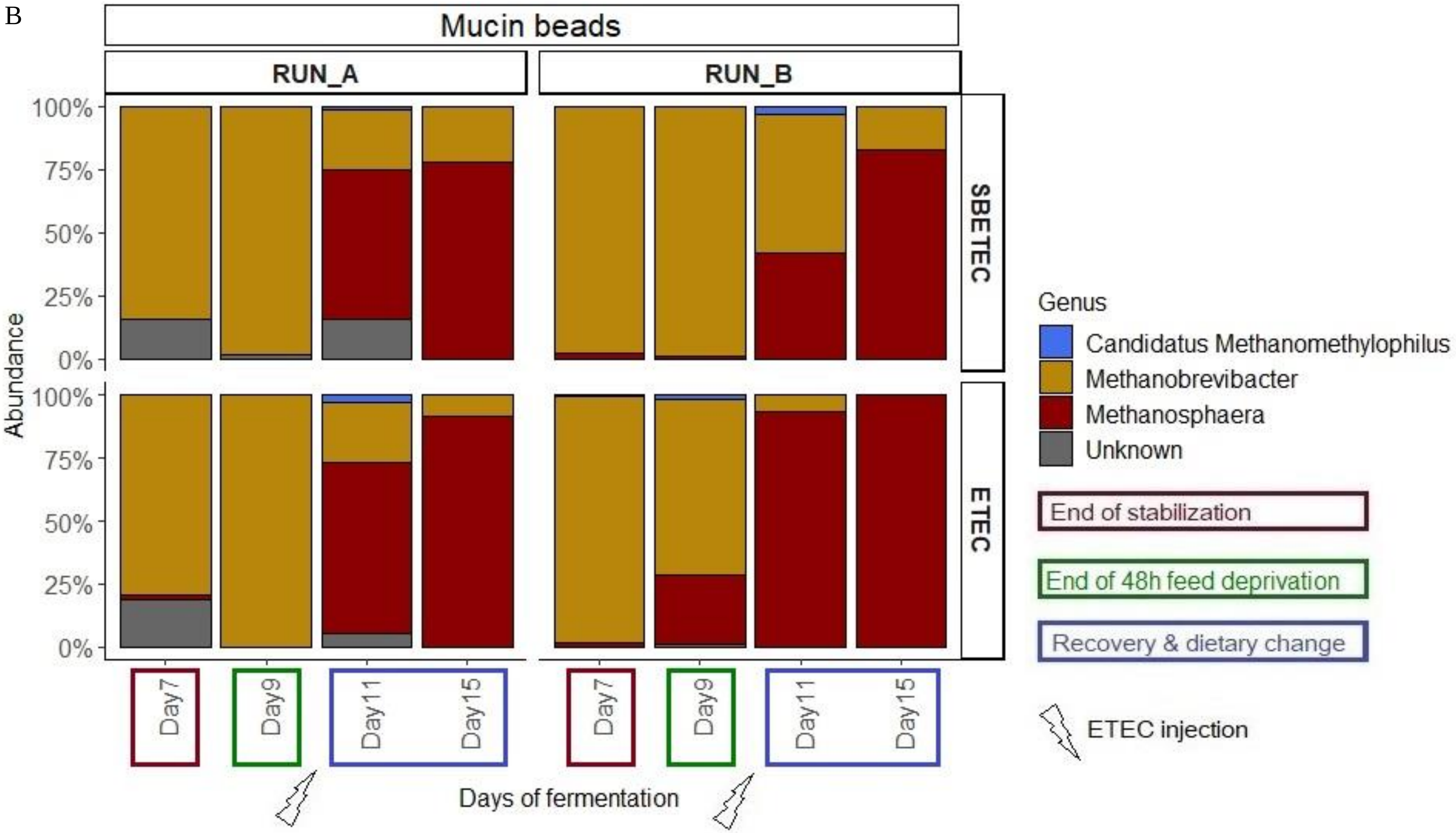

Mucin beads
RUN_A
RUN_B
SBETEC
ETEC
100%
75%
50%
25%
0%
Abundance
Day7
Day9
Day11
Day15
Days of fermentation
Genus
Candidatus Methanomethylophilus
Methanobrevibacter
Methanosphaera
Unknown
End of stabilization
End of 48h feed deprivation
Recovery & dietary change
ETEC injection

Supplementary Figure S6. Quantification of bacterial and archaeal groups by qPCR in the bioreactor medium (A), mucin beads (B) and bead medium (C) of the MPigut-IVM during the ETEC and SBETEC conditions (n =2).

A

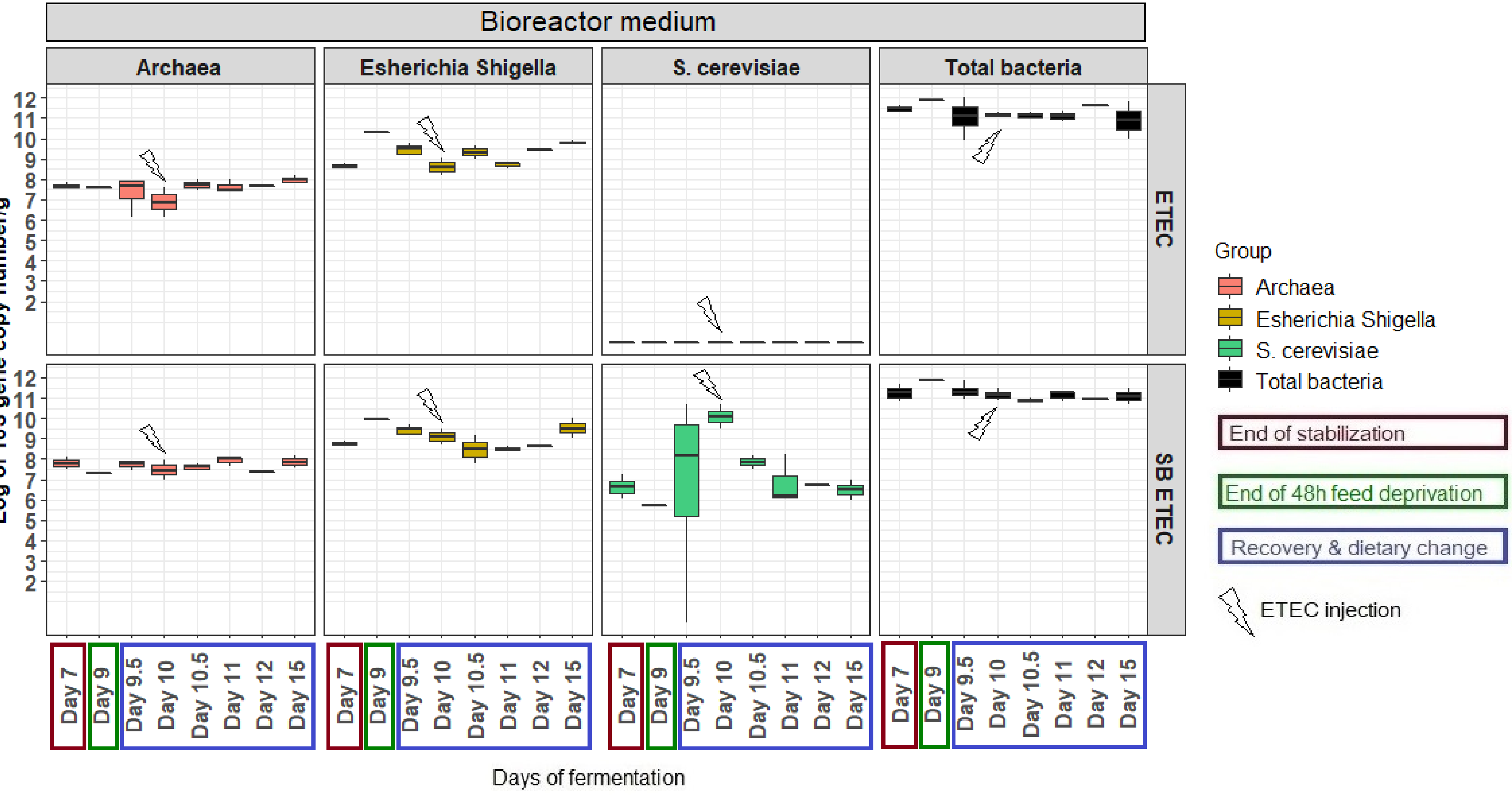

B

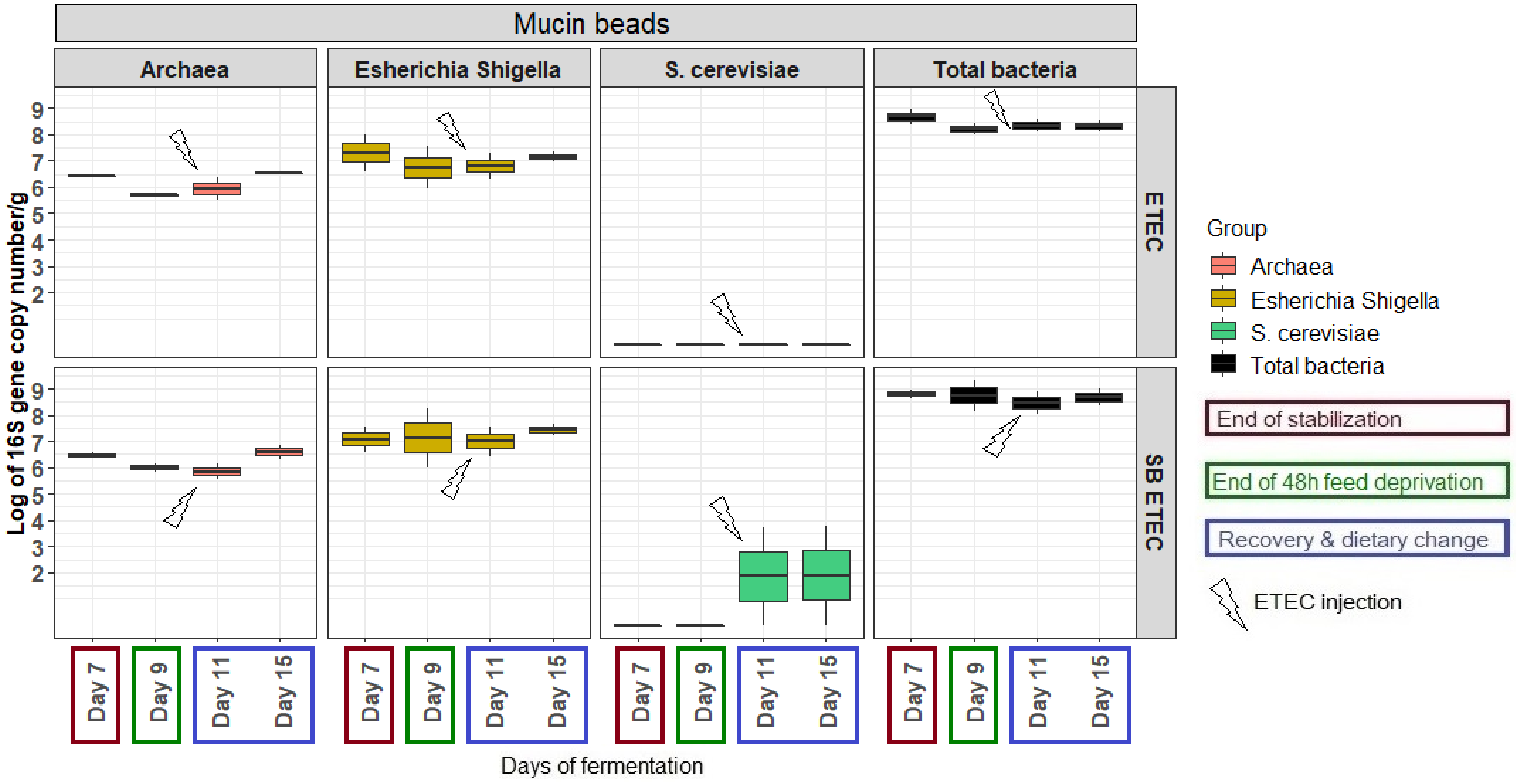

Mucin beads
Archaea
Esherichia Shigella
S. cerevisiae
Total bacteria
ETEC
SB ETEC
Log of 16S gene copy number/g
9
8
7
6
5
4
3
2
Day 7
Day 9
Day 11
Day 15
Days of fermentation
Group
Archaea
Esherichia Shigella
S. cerevisiae
Total bacteria
End of stabilization
End of 48h feed deprivation
Recovery & dietary change
ETEC injection

C

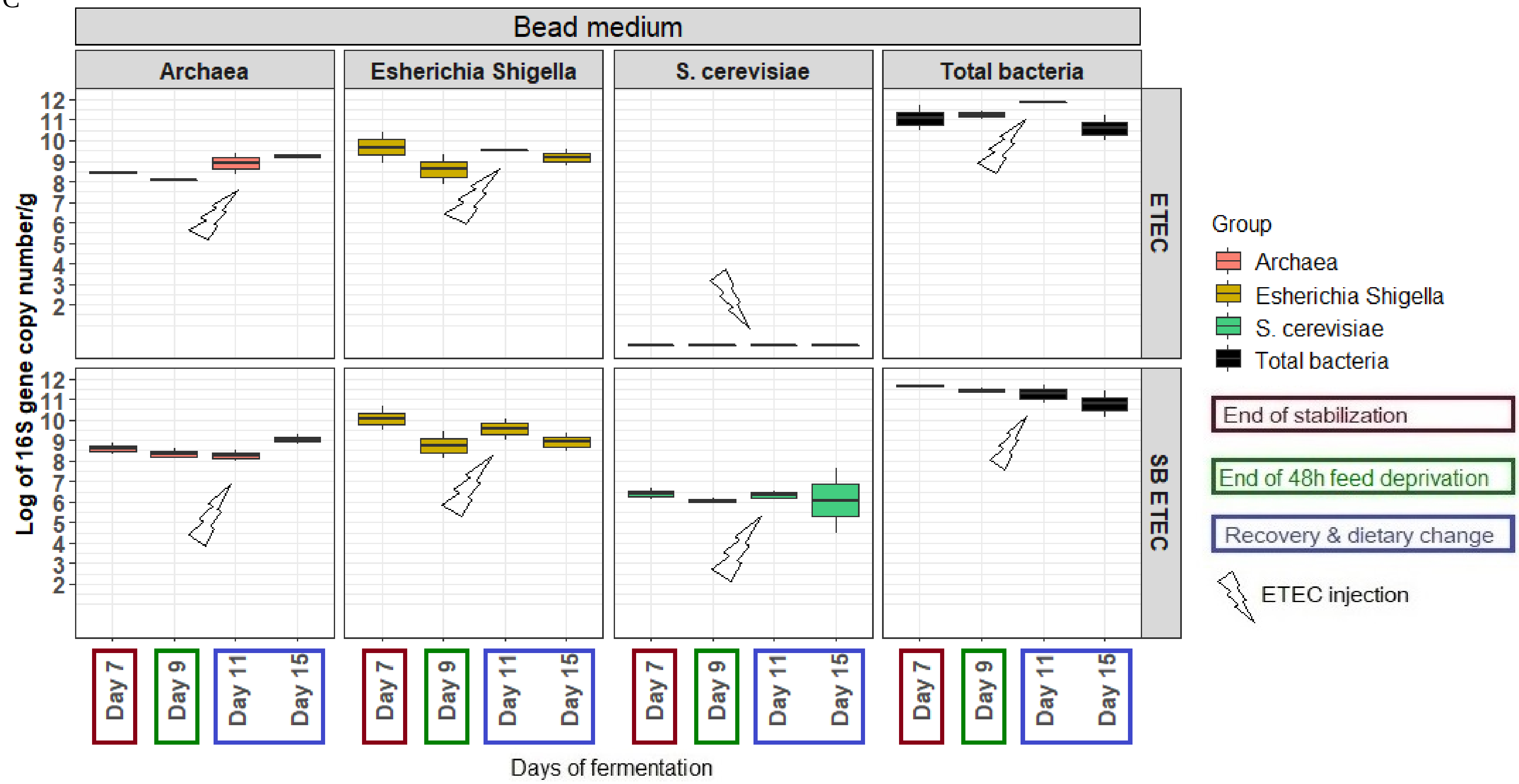

Bead medium
Archaea
Esherichia Shigella
S. cerevisiae
Total bacteria
ETEC
SB ETEC
Log of 16S gene copy number/g
12
11
10
9
8
7
6
5
4
3
2
Day 7
Day 9
Day 11
Day 15
Days of fermentation
Group
Archaea
Esherichia Shigella
S. cerevisiae
Total bacteria
End of stabilization
End of 48h feed deprivation
Recovery & dietary change
ETEC injection
